# Mesoscale Soft Robotics for Mechanically Perturbing and Functionally Monitoring Responses in Human Organoids

**DOI:** 10.64898/2026.10.28.762586

**Published:** 2026-10-30

**Authors:** Woo-Youl Maeng, Zengyao Lv, Yasaman Samei, Kyoung-Ho Ha, Kyung Su Kim, Lakshmi P. Prabakar, Ivana A. Chychula, Vasant Iyer, Binjie Li, Yuxuan Mao, Shengwei Xu, Tae Wan Park, Shupeng Li, Cory W. Holgren, Young Jin Jo, Hak-Young Ahn, Naijia Liu, Seonggwang Yoo, Seyong Oh, Jahyun Koo, Elizabeth M. McNally, Igor R. Efimov, Dominic E. Fullenkamp, Yonggang Huang, John D. Finan, John A. Rogers

## Abstract

Human organoids represent promising models for studies of significance to the biology and medical communities. Of the existing approaches for monitoring organoids, none offers the ability to apply programmable mechanical forces while simultaneously recording multi-modal physiological responses, of relevance to phenomena spanning from traumatic brain injuries to cardiac rhythm disorders. Here, we present a mesoscale soft robotic organoid interface that enables programmable delivery of forces to organoids of diverse shapes, and to map electrophysiological activity and mechanical responses. The platform combines actuation elements with integrated sensors for quantitative measurement and control. Studies using this technology reveal that spheroidal cortical organoids exhibit distinct responses to mechanical trauma, including silencing, recovery, and changes in neuronal firing patterns. Additional experiments on cardiac ring tissues demonstrate that sustained stretching induces beat-to-beat rhythm variability and altered conduction. The results establish a framework for investigating force-triggered transitions between physiological and disease-like states in human tissue models.

**HIGHLIGHTS:**

A mesoscale soft robotic interface delivers programmable forces to human organoids
Versatile geometries deliver compression, stretch, and shear to varied tissue shapes
Real-time recording captures trauma-induced silencing and recovery in organoids
Multimodal readout tracks rhythmic changes in engineered heart tissue under loading

## Full Text Availability

The license terms selected by the author(s) for this preprint version do not permit archiving in PMC. The full text is available from the preprint server.

